# Social Environment Affects Acquisition and Color of Structural Nuptial Plumage in a Sexually Dimorphic Tropical Passerine

**DOI:** 10.1371/journal.pone.0047501

**Published:** 2012-10-17

**Authors:** Rafael Maia, Luiza Brasileiro, Roberto V. Lacava, Regina H. Macedo

**Affiliations:** 1 Programa de Pós-Graduação em Ecologia, Universidade de Brasília, Brasília, DF, Brazil; 2 Department of Biology, Integrated Bioscience Program, The University of Akron, Akron, Ohio, United States of America; 3 Laboratório de Comportamento Animal, Departamento de Zoologia, Universidade de Brasília, Brasília, DF, Brazil; University of Sussex, United Kingdom

## Abstract

Structural colors result from the physical interaction of light with organic materials of differing refractive indexes organized at nanoscale dimensions to produce significant interference effects. Because color properties emerge from these finely organized nanostructures, the production of structural coloration could respond to environmental factors and be developmentally more plastic than expected, functioning as an indicator of individual quality. However, there are many unknown factors concerning the function and mechanisms regulating structural coloration, especially relative to social environment. We hypothesized that social environment, in the form of competitive settings, can influence the developmental pathways involving production of feather structural coloration. We experimentally assessed the impact of social environment upon body condition, molt and spectral properties of two types of structural color that compose the nuptial plumage in blue-black grassquits: black iridescent plumage and white underwing patches. We manipulated male social environment during nine months by keeping individuals in three treatments: (1) pairs; (2) all-male groups; and (3) male-female mixed groups. All morphological characters and spectral plumage measures varied significantly through time, but only acquisition of nuptial plumage coverage and nuptial plumage color were influenced by social environment. Compared with males in the paired treatment, those in treatments with multiple males molted into nuptial plumage faster and earlier, and their plumage was more UV-purple-shifted. Our results provide experimental evidence that social context strongly influences development and expression of structural plumage. These results emphasize the importance of long-term experimental studies to identify the phenotypic consequences of social dynamics relative to ornament expression.

## Introduction

Integumentary color is widespread in animal taxa and functions within various contexts, ranging from protective effects, for example as countershading or disruptive coloration [1] to advertisement in the attraction of prospective mates [2]. There is presently a good deal of interest concerning the mechanisms involving the production of color (structural versus pigmentary) and the different costs and benefits associated to these forms of color expression. Sexual signaling based upon plumage color, particularly relative to pigmentary colors, has been especially emphasized. However, fewer studies have addressed both the dynamics of sexual trait expression and its context of occurrence, whether social or ecological, to adequately test hypotheses of condition-dependence and honest signaling [3], [4].

Pigmentary coloration has been more intensively studied than structural coloration, and within the former, studies concerning colors based on carotenoid pigments have been particularly numerous. It has been suggested that carotenoid coloration represents a trade-off between immune function and sexual signaling [5], and could thus ensure the maintenance of honest male signaling in several taxa. For example, carotenoid-based beak color in some birds has been found to dynamically reflect dietary content [6] or social status [7]. Carotenoid-based ornaments are also suggested to reflect stress or social rank in organisms as diverse as lizards [8] and fish [9]. Thus, ornamental carotenoid-based coloration of feathers or integument in a diversity of animal taxa can indicate the health of the individual [10] or level of stress relative either to the social or physical environments [11], [12], [13].

Structural coloration is broadly represented in metazoans [14], [15], being widespread and extremely diverse in birds [16]. However, in sharp contrast with the abundant evidence for carotenoid-based colors, the condition dependence of structural coloration has been little studied at both the proximal and functional levels [17], [18]. Structural colors are produced through the physical interaction of light with organic surfaces of different refraction indexes [19], [20]. Most short wavelength colors in animals are structural, and include green, blue and ultraviolet colorations [19]. Depending upon the light-scattering mechanism, structural colors can also be iridescent, so that their appearance changes depending upon the angle of observation or incidence of light [21]. The nanometer-scale variation in shape, size and organization of structures responsible for structural coloration of feathers (mainly keratin, melanin and air) can produce a large assortment of colors, as seen in the iridescent colors of hummingbirds [22]. Although the exact ontogenetic mechanisms that produce structural coloration are still not fully established (but see [23], [24], [25]), some studies have suggested a functional role for structural colors as signals of individual quality [26], [27], as well as its condition dependence [18], [28], [29], [30], [31].

Feather structural colors may be regulated at a proximate level [31], [32], [33], with a possible role for external environmental agents, including parasites [29], [34], [35], [36] and nutritional deficiencies [18], [28]. However, most of the collected evidence suggesting condition dependence of structural plumage color has been correlational [19]. Additionally, recent experimental evidence has challenged the direct link between diet quality and structural color, highlighting instead that such colors may be more sensitive to environmental stressors [37]. Male variation in ornamentation, though commonly seen as resulting from feedback mechanisms based upon individual internal condition, is increasingly being accepted to also derive from extrinsic dynamic interactions with conspecifics of both sexes [38].

We experimentally tested the hypothesis that social context influences the molt and expression of ornamental structural colors of birds using a captive population of blue-black grassquits (*Volatinia jacarina*; Linnaeus, 1766), a common Neotropical small passerine [39]. Populations of blue-black grassquits migrate into central Brazil during early November to breed, at which time males molt from their unobtrusive brownish female-like plumage into a blue-black iridescent plumage with conspicuous white underwing patches [39], [40]. Observational field evidence indicates that the quality and color of the iridescent nuptial plumage of breeding males may reflect parasitic load [35], nutritional condition [28] and investment in molt [41]. The nuptial plumage is exhibited during the sexual display, which consists of repeated, short vertical flights from perches at the periphery of the males' small territories [40], [42], [43]. Though socially monogamous, the high rate of extra-pair fertilizations [44], associated with the lek-like clustered territories [45], [46] highlight the importance of social interactions and the strong intra- and intersexual pressures that shaped this species' mating system [47]. Females nest within male territories, and both sexes feed the offspring [43]. The sexual signaling involving plumage and behavior as well as the mating system of the blue-black grassquit make it an excellent model species to investigate the role that social context plays in the expression of ornamental traits.

The blue-black grassquit exhibits two types of structurally-colored feathers: the black iridescent coverage is produced by an organized array of melanin granules that define a keratin thin-film [48]; and the white underwing patch is commonly produced by an unorganized matrix of keratin [49]. Therefore, though both colors observed in the nuptial plumage of male blue-black grassquits are structural, one of them derives from the ontogenetic nanostructural organization of biomaterials, while the other does not. These plumage characteristics provide a unique and ideal system to disentangle the susceptibility of the potentially costly production of feather materials and its subsequent developmental organization to extrinsic factors. We also examined how different plumage and body condition variables varied through an extended time period that included the breeding season. We subjected males to three social arrangements to create different levels of social competition and interactions: (1) pairs composed of one male and one female; (2) all male groups composed of six males; and (3) mixed groups composed of three males and three females. Accordingly, we predicted that males molting in a low competitive social environment (paired male with female) would exhibit a lower investment in the quality of their nuptial plumage compared to males in more competitive social contexts (mixed groups and all males groups). Within the latter, we predicted that males in mixed groups would invest more heavily than those in the all males groups, due to possibly more rigorous competitive interactions for access to females. We expected a high investment to be characterized by faster molting, more complete nuptial plumage coverage, and/or higher spectral measures associated with brightness and saturation.

## Materials and Methods

### Ethics statement

All procedures were authorized by CEMAVE (license no. 1301/2) and Instituto Brasileiro do Meio Ambiente e dos Recursos Naturais Renováveis (IBAMA license no. 206/2006).

### Study animal treatment and housing

We captured 46 male and 22 female blue-black grassquits within the campus of the University of Brasilia, Brazil (15^°^ 46′S; 47° 52′W) between February and March 2007, marked them with numbered metal bands (Centro Nacional de Pesquisa para Conservação das Aves – CEMAVE) and kept males and females visually isolated from each other within subdivisions of an outdoor aviary (5 m ×10 m ×3 m) until June 2007. The outdoor aviary exposed the birds to the normal photoperiod of the area, and they presumably maintained a reproductive physiology similar to that of wild birds. All birds were treated with vermifuge (mebendazole) and medication for ectoparasites (sulphametoxazol–trimethoprim, pirethrin talc), and provided with an *ad libitum* diet of green, red and yellow millet and birdseed, in addition to vitamin-fortified water and sterilized sand. Experiments were conducted in the Animal Behavior Laboratory of the University of Brasilia from July 2007 to July 2008.

### Experimental design

Birds were randomly divided into three social treatments in July 2007: (1) pairs of one male and one female (“paired”); (2) mixed groups of three males and three females (“mixed”); and (3) all-male groups of six males (“all males”). Each social treatment had three replicates, each of which was housed in a large compartment (2.6 m ×1.5 m ×2 m) while the pairs were kept in cages (0.55 m ×0.30 m ×0.38 m), all within the outside aviary. The size differences between compartments for the group treatments versus pairs provided more space for individuals within groups, and this may have resulted in some unidentified behavioral effects. However, we believe that the higher space allotment for groups counterbalanced the much higher number of possible aggressive pairwise interactions that could occur among the six individuals. Males in captivity have a reduced rate of display leaps as compared to those in natural conditions, but exhibit similar leap heights of about 32 cm (Carvalho et al. 2007; Aguilar et al. 2008). Thus all captivity conditions used, even the smaller cages where paired males were kept, allowed conditions for leaping at maximum heights, although the display was infrequently observed. All compartments and cages were located in an outside aviary, thus exposed to natural light conditions, and had visual but not acoustic isolation. Each treatment also had a backup replicate kept in similar housing and overall conditions to substitute the experimental replicate in case needed. We collected data monthly from all males from August 2007 until July 2008 that included morphometric, plumage coverage as well as spectral and color measures of the plumage (see below). In a parallel study, we also quantified testosterone plasma concentration and aggression levels of males in the mixed and all males treatments to assess the intensity of social competition [50]. Given the associated variation found for testosterone levels with time and the experimental treatments, direct tests of the association of hormonal levels with the phenotypic variables herein measured would be confounded and therefore we restricted ourselves to the discussion of their associated trends.

### Morphological and plumage coverage measures

Each month we collected data on: (1) body mass index; (2) percent nuptial plumage coverage, estimated from the average proportion of 35 equidistant dots in a 3 cm transparent disk covered by nuptial plumage when placed over the head, breast and rump, from a different area than that being sampled for feathers (see below), modified from [35], [51]; and (3) relative area of white underwing patch taken as the area of the patch divided by the total wing area from the tracing of the wing under an acetate sheet, averaged from both wings [35], [41], [51]. We measured the digitalized (300 dpi) wing drawings using a threshold procedure in Image J v.1.38 [52].

### Spectral measures and color properties

Each month 3–6 blue-black rump and white left wing patch feathers were collected by the same person to analyze spectral properties of the plumage; these were assigned unique individual identity numbers that did not refer to the experimental treatment conditions. The rump area was chosen for its putative signaling role [28], as well as for being a protected area of the body, thus minimizing any increased effect of abrasion due to captive conditions – as were the white feathers, collected from underwing patches. During sampling, additional feathers in the area were removed, marking a clear molting area from which new feathers could be identified and plucked, ensuring that the feathers collected over several months had grown between samplings and reflected the recent conditions experienced by that individual. During this and previous experiments [25] we observed that feather replacement took between 2 and 4 weeks, thus the monthly sampling allowed us to collect full-grown feathers while the sampling area could be identified through several partially-molted feathers. We observed that the replacement of white patch feathers seemed to be slightly slower than that of black ones. Further, in the first month, some males had not yet initiated molt in the rump area, and therefore were not sampled.

Three of the feathers collected each month were taped in an overlaid pattern on a black velvet substrate, simulating the pattern on the bird's body, and their reflectance was measured with an Ocean Optics USB4000 spectrometer attached to a pulsed xenon light source (range 250–750 nm; Ocean Optics PX-2, Dunedin, FL). All measurements were taken using unpolarized light and relative to a WS-1-SS white standard (Ocean Optics). For all reflectance measurements we used two separate optic probes (UV-VIS, core diameter 400 μm), one attached to the light source and the other to the spectrometer, held specularly at 45° from the surface, the normal measurement geometry in which most repeatable values are obtained for this species [41] at approximately 6 mm from the sample surface, held by a block sheath that excluded ambient light. We used SpectraSuite software (Ocean Optics) to take three measurements of all samples, each calculating an average of 50 sequential spectra recordings. Between sampling events we lifted the probe from the feather sample to guarantee representative measurements of the feather. All measurements were interpolated to a step width of 1 nm and calculations were performed based on the average spectra of these three repetitions, from 300 to 700 nm, the approximate visible spectra of birds [53], [54]. For the analyses of the achromatic white patch feathers we used only measures of brightness and UV chroma [41], [55].

Given that coloration properties derived from spectral measurements are commonly correlated, we conducted a principal component analysis (PCA on correlation matrices, no factor rotation) on data from individual average reflectance spectra of the iridescent plumage [56], [57], [58]. Reflectance values for each spectrum were binned to 20 nm intervals across the measured spectrum, and the resulting 20 values were included in the PCA. To ease interpretation of the resulting components, colorimetric variables (following [58]; to which variable names refer) were calculated and correlated to the new variables.

**Figure 1:**
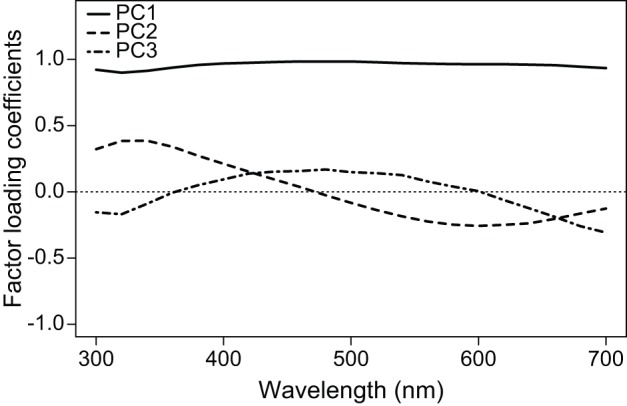
Principal component analysis of iridescent feather color. Factor loading coefficients for the first three principal components extracted from the nuptial plumage reflectance spectra of male blue-black grassquits in relation to wavelength.

The first component (PC1) had high and positive factor loadings for all wavelengths, and was therefore taken as an estimate of achromatic reflectance, or average brightness. PC2 loaded highly for both short and long wavelengths, but in opposite directions, and was strongly negatively correlated to hue, strongly positively correlated to UV chroma, and negatively (but relatively weakly) correlated to blue chroma and positively correlated to UV chroma. Therefore, we interpreted PC2 as a measure of hue, and the consequent increase and decrease of the proportion reflectance in the UV and blue areas as the spectrum's peak shifts along those wavelengths. Finally, PC3 had loadings that were higher and positive for intermediate wavelengths, and correlated strongly and positively to blue chroma, with weak (but significant) positive correlation with hue and negative with UV chroma. Thus, being orthogonal to PC1 and PC2, we interpreted PC3 as a measure of level of concentration, or saturation, of the reflectance in the blue region; high values indicate reflectance concentrated in the blue region, and low values indicate proportionally more reflectance in the UV-violet and red areas, producing violet-purple colors. Together, the first three principal components accounted for 99.3% of total and 91.3% of chromatic (after accounting for brightness) variation in reflectance ([57]; Fig. 1 and table 1).

**Table 1:** Correlation of descriptive color variables with the principal component scores.

Variable	*r*		
	PC1	PC2	PC3
Average brightness (B1)	*0.99*	0.05	0.04
Peak reflectance intensity (B3)	*0.98*	*0.17*	0.11
Hue (H1)	0.12	*−0.62*	*0.44*
UV chroma (S8; 300–400 nm)	−0.01	*0.93*	*−0.23*
Blue chroma (S8; 400–500 nm)	*0.21*	0.17	*0.84*
Variance explained (total/chromatic)	91.9%	5.3%/64.9%	2.1%/26.4%

Chromatic variance refers to the variation explained by the component after accounting for the achromatic (brightness) component; variable names follow [58]. Italicized values: P<0.01 (after Bonferroni correction for multiple comparisons).

### Statistical analysis

Since color properties and other phenotypic traits could vary nonlinearly with time due to the seasonal aspects of blue-black grassquit reproduction, we used a generalized additive mixed model (GAMM) approach to our analysis, since it allows for the model fit to include both parametric terms and non-parametric smoothers, as well as random effects to account for data non-independency [59]. For each model, the morphological and color trait of interest was included as the response variable, social treatment group as a fixed effect, and time as a penalized regression spline, with maximum likelihood estimated smoothed parameters [59]. Since the response variable could potentially vary differently through time for the different groups (a “treatment-by-time interaction”), we used varying-coefficients models [60], which allow for smooth terms to be fit conditional to the parametric variable, thus producing a treatment-specific temporal response. Significance of terms was assessed through backward stepwise procedures, in which covariates were removed (starting with higher-order interactions) from the full model and compared to it through likelihood ratio tests (LRT), using the difference in deviance as a chi-squared approximation [59]. Given our experimental design, possible sources of non-independence in our models included the repeated measures of individual birds, and caging conditions for the group treatments [50]. Therefore, we included individual identity nested within cage identity as random effects in all our models.

**Figure 2:**
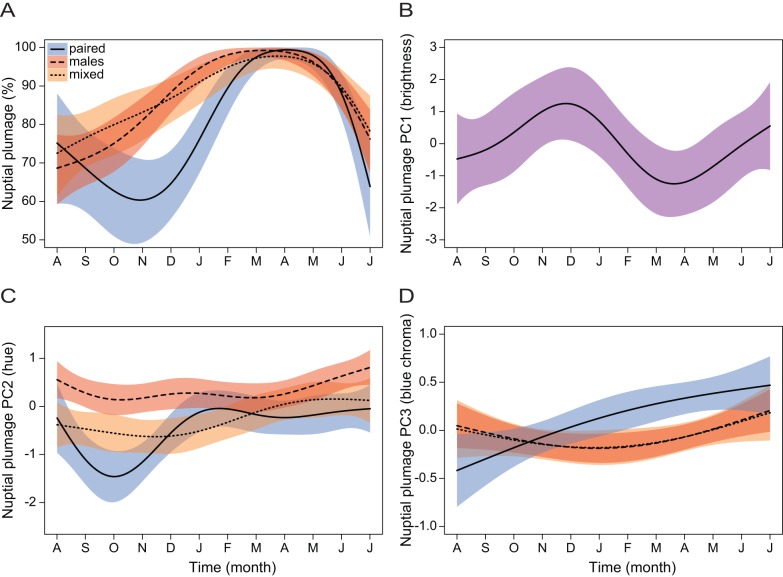
Social environment and time affect the expression of iridescent nuptial plumage in male blue-black grassquits. Variation across nine months (November 2007– July 2008) of (**A**) percent coverage, (**B**) color PC1 (brightness), (**C**) PC2 (hue) and (**D**) PC3 (blue chroma) of the iridescent nuptial plumage of male blue-black grassquits. Effects are shown pooled (violet shading) across treatments when there were no treatment or interaction effects, as the simplified model was used for inference; shadings represent 95% confidence intervals.

All analyses were conducted in R v.2.15.1 [61] with packages mgcv v.1.7–19 and gamm4 0.1–6 [59]. Though we initiated our experiments with 36 males (9 paired males, 6 males in each of three all-male groups, and 3 males in each of three mixed groups), 10 males died in the first three months of the experiment [50]. These individuals were replaced by males being kept in backup groups maintained under the same three treatment conditions. We conducted the analyses considering all individuals, although analyses considering only the period after which replacements occurred (from November 2007 to July 2008; [50]) yielded the same results. To ensure treatment effects did not reflect differences between groups at the start of the experiment, we conducted Analyses of Variance (ANOVA) for all variables for their values in the first month. We took a conservative approach and did not correct for multiple comparisons in these tests. None of the morphological variables showed differences in the first month (all p>0.05), but relative to the colour variables PC2 showed a significant difference between treatments (F_2,29_ = 4.12, p = 0.03; see Fig. 2C), with males from the all males group showing higher values than the mixed group, but no differences between the mixed an paired nor the paired and all males groups (pairwise comparisons using Tukey's Honest Significant Difference test; see Figure S1). Thus, results based on the analysis of this variable should be considered cautiously due to these initial differences.

## Results

We found a significant interaction effect of time and treatment on the percentage of blue-black plumage coverage, indicating that molt patterns were not uniform for the males in different social contexts (Table 2; Fig. 2A). Males in both the all males and mixed treatments achieved their peak nuptial plumage coverage earlier (with the all males treatment being the earliest) and maintained an overall higher coverage compared with males in the paired treatment throughout most of the experiment. Males from the paired treatment continued exhibiting their cryptic plumage for a few months after the beginning of the experiment, only initiating full nuptial molt in December, after four months exposed to experimental conditions. Nuptial plumage brightness (PC1) did not vary between treatments (Table 2), but showed a sinusoidal pattern of variation through time, with lowest values achieved between February and May – precisely when nuptial plumage coverage was at its peak (Fig. 2A, B). There was a significant interaction effect in PC2, which mostly reflected the initial higher value for individuals in the all males group. Though these individuals maintained relatively higher values throughout the experiment, these differences gradually diminished and all groups showed similar values, with slight temporal oscillations, throughout most of the experiment. Finally, we found an interaction between social environment and time for the blue chroma (PC3; Table 2). Whereas the amount of reflectance concentrated in the blue region increased approximately linearly over time for males in the paired treatment, males in both social treatments (all-males and mixed) showed overall lower values for this variable throughout the experiment, declining slightly and with a concave temporal pattern (Fig. 2D). In other words, males from the paired treatment exhibited a more blue-shifted coloration, which became bluer over time, whereas males from the mixed and all males treatments displayed a more violet-UV-shifted coloration.

**Table 2:** Generalized additive mixed models testing the effects of social treatment group and time upon body condition and plumage attributes.

	Time			Treatment			Time X Treatment		
Variable	*χ^2^*	Df	P	*χ^2^*	df	P	*χ* ^2^	df	P
Body mass	95.87	5	*<0.001*	4.56	2	0.10	12.61	10	0.25
Underwing patch relative area	17.74	2	*<0.001*	0.31	2	0.85	4.28	4	0.37
Patch brightness	55.83	5	*<0.001*	3.85	2	0.15	8.54	10	0.58
Patch UV	38.58	4	*<0.001*	0.06	2	0.97	15.15	8	0.06
% nuptial plumage	208.36	4	*<0.001*	0.61	2	0.74	*32.51*	*8*	*<0.001*
PC1	15.03	5	*0.01*	0.41	2	0.81	5.45	10	0.86
PC2	35.82	4	*<0.001*	7.50	2	0.02	18.89	8	0.02
PC3	13.94	2	*<0.001*	*2.51*	*2*	*28*	10.16	4	0.03

See “Methods” for details of the statistical analysis.

**Figure 3:**
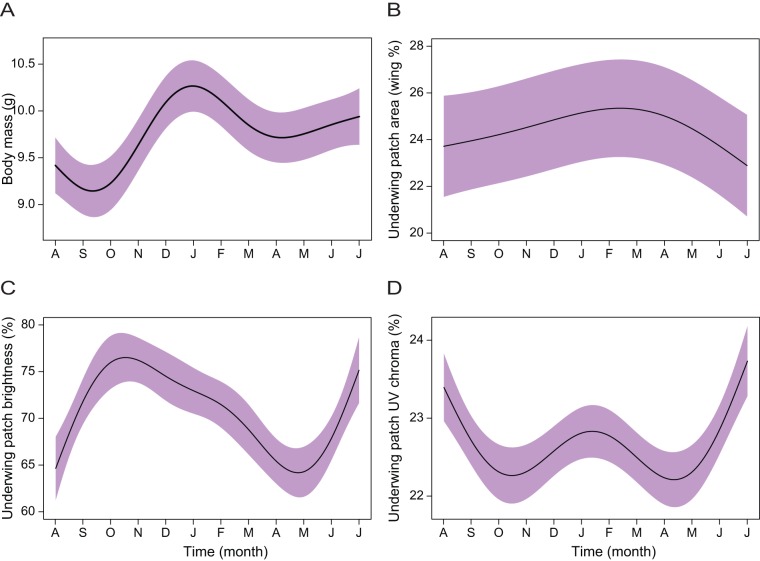
Time affects body condition and white underwing patch expression in male blue-black grassquits. Variation across nine months (November 2007 – July 2008) of (**A**) body mass, and (**B**) white underwing patch relative area, (**C**) brightness and (**D**) UV chroma of male blue-black grassquits. Effects are shown pooled (violet shading) across treatments when there were no treatment or interaction effects, as the simplified model was used for inference; shadings represent 95% confidence intervals.

Male body mass varied significantly over time but not between treatments (Table 2), with an overall increase over time with a marked peak between December and February (Fig. 3A). The relative size of the white underwing patch also showed a slight variation through time (Table 2), with a gradual increase during the breeding period (between January and May), and a steady decline afterwards (Fig. 3B). Also during this period, underwing patch brightness presented its lowest values, after peaking in October, just before the breeding season (Table 2; Fig. 3C). Underwing patch UV chroma values declined markedly before and after the breeding period, with a peak during the breeding period, though values at the start and end of the experiment were similarly high (Fig. 3D). These temporal patterns were consistent across social treatments for all of these variables (Table 2).

## Discussion

### Nuptial plumage differences between treatments

We found that beyond the typical variation in plumage characteristics that occurs in cyclical fashion and associated with seasonality, social context strongly influenced molting patterns in the blue-black grassquit. In the two treatments where males interacted continuously (all males and mixed treatments), molting into the nuptial plumage occurred at a faster rate, reached maximum values earlier, and these males maintained higher nuptial plumage coverage through time (Fig. 2A). These males also molted to a nuptial plumage with reflectance less concentrated in the blue region, i.e. with relatively more reflection in both short and long wavelengths, thus producing colors with a purple iridescent sheen (Fig. 2D). However, there was no discernible difference in the overall plumage pattern between males in the all males versus the mixed treatment. Paired males, on the other hand, stayed brown for a longer time period, and though they reached similar peak levels of nuptial plumage coverage, they took longer to do so and remained in that plumage for a considerably shorter time. The plumage of paired males was concentrated in mid-range wavelengths, i.e., blue-shifted. Our results suggest that in treatments in which males interacted with other males, individuals initiated molting into a more purplish nuptial plumage quickly, and maintained it for a longer time compared with males deprived of contact with other males. It thus seems reasonable to assume that in the case of the paired males, the tardy investment in nuptial molting may have occurred because they were not exposed to possible rivals.

Two main hormones regulate the sexual dichromatism of color in most passerines: androgens and luteinizing-hormone (LH) [62]. Testosterone appears to be the main link between a male's condition and level of sexual signaling, as suggested in a study with the superb fairy-wren, *Malurus cyaneus*, which found a positive relationship between testosterone level and molt, as well as in the maintenance and brightness of structural coloration [32]. However, the mode of action of testosterone remains unclear, though it has been suggested that this hormone triggers molting, and in particular prenuptial molt, but other factors could determine plumage coloration [32], [33].

In a parallel study with the same group of blue-black grassquits, we quantified testosterone levels and aggressive interactions according to social environment [50] and found a significant variation in testosterone levels through time, peaking in the breeding season (between February and May), as could be expected. But more importantly, we also found that the patterns of testosterone variation through time differed greatly among treatments, with paired males presenting a lower and delayed testosterone peak relative to the treatments where males were interacting with other males. We also found that there were more aggressive interactions in the all males treatment when compared with the mixed treatment. Thus, results relative to the nuptial molting patterns agree with hormonal and behavioral responses, all of which suggest that the physiological and phenotypic response of males may reflect a first reaction to competition with other males, whether or not females are present. Further, previous experiments with blue-black grassquits have shown that nuptial plumage cover mediates aggression, with males with a more developed plumage being targeted with more aggression in dyadic encounters [63]. In other words, hormonal levels and social interactions likely interplay dynamically [50], affecting the expression of ornamental traits such as the prenuptial molt in blue-black grassquit males, which can be assessed by females and other males. These results clearly illustrate the scenario recently envisioned by Rubenstein and Hauber [38], wherein the development and maintenance of sexual ornaments should be deeply dependent upon the interplay between physiological, morphological and behavioral costs.

Interesting patterns are also evident when we compare temporal responses of the different phenotypic traits herein considered. Several traits showed dramatic variations associated with the months of March through May. This period coincides with the end of breeding, and for all treatments we found that nuptial plumage coverage was at its peak while both the iridescent and underwing patch coloration showed a marked decrease in brightness, and at a smaller scale, so did body condition. These results suggest a possible trade-off between the acquisition of the nuptial plumage and its coloration attributes, as well as a declining response to the need for sexual signaling.

A previous field study with this species concluded that the investment in nuptial plumage is positively associated to chromatic, but not achromatic, properties of coloration [41]. Consistent with those results, here we found that both the acquisition of nuptial plumage and its chromatic (but not achromatic) properties possibly represent a socially-mediated response in ornamental investment. In blue-black grassquit iridescent feathers, color is produced by a single thin-film of keratin over a melanin layer [48]. In thin-film colors, chromatic properties derive from the nanostructural arrangement of melanosomes and keratin within feather barbules, whereas brightness possibly results from overall feather condition or number of barbules [64]. It is thus likely that producing feathers with certain nanostructure-associated attributes (such as a saturated, short-wavelength-shifted color) represents a testosterone-mediated trade-off [65], [66]. On the other hand, achromatic properties – both of the iridescent plumage and the white underwing patch – showed a temporal decrease that coincides with the testosterone peak found in the parallel study with the same birds [50].

### Variation of body condition and plumage variables through time

All six plumage variables as well as body mass in the three experimental treatments displayed temporal variation, illustrating the seasonal profile of energy acquisition and investment prior to and during the breeding period. During the molting period many small passerines exhibit abrupt temporal changes in body mass. In some species body mass decreases during molt, presumably to increase flight ability [67], [68]. In other species, however, body mass increases [32], which may be due to the high energy demand associated with feather replacement [69], [70]. Thus, given the hormonal profile of this captive blue-black grassquit population, it is not surprising that body mass displayed a slight oscillation, peaking at the onset of the breeding period, wherein activities typically consume a high level of energy (e.g. prenuptial molt, territory acquisition), with a subsequent decline. However, our data suggest that factors other than body mass index are the main cause of variation in acquisition of nuptial plumage, since we only found a significant interaction between time and treatment for the latter.

Until now we had few clues about the signaling function of the white underwing patches of the blue-black grassquit. These patches are exhibited repeatedly during the leaping sexual displays, suggesting that this trait is important in a sexual context [51]. However, results from past studies failed to find a relationship between size or color of the white patch and parasitism, body condition, or molting conditions, despite the impact of both parasitism and molt upon the blue-black nuptial plumage [35], [41], [51]. These previous studies, however, were conducted during the few months of this species' breeding season. Here we demonstrate that the relative size of the white patch as well as its brightness vary throughout the year, reaching their highest values during the breeding period (Nov.-Mar.). The white patch is an important visual component of the leap display, in which the repeated beating of the wings exposes the patch as a flashy achromatic signal that contrasts markedly with the blue-black iridescent body plumage. Such signals have been shown to be particularly effective in long-distance communication [71], [72]. The benefits of producing an increasingly conspicuous underwing plumage color may thus be crucial during the breeding season to establish a territory and/or attract a mate. In this context, our results indicate that although the white underwing patches may not be condition-dependent [35], [41], [51], their expression is still enhanced during the period in which they are exhibited in leaping displays. It is likely that the patches function as a signal component to amplify the conspicuousness of the motor display and the contrasting blue-black iridescent plumage [73], [74].

### Conclusions

The observed patterns of structural nuptial plumage variation in the blue-black grassquit suggest that both the social context as well as seasonality exert strong pressures in ornamental expression. Indeed, we believe that this is the first study to show an effect of social context upon the acquisition of structurally colored plumage. Both intra and intersexual contexts appear to influence the acquisition and quality of the nuptial plumage in this species, with more competitive contexts leading to an early acquisition of iridescent blue-black plumage. We suggest that aggressiveness and testosterone jointly contribute to the acquisition and color properties of male nuptial plumage in this species, and possibly others with similar characteristics. Additionally, we also found that although the white underwing patches exhibited during displays do not appear to be condition-dependent, this trait potentially plays a critical role in sexual signaling by amplifying the conspicuousness of both the motor and color aspects of the display. The white patch shows a temporal trend, with relative size and brightness peaking during the breeding period and declining quickly afterwards.

We emphasize the importance of long-term and experimental studies of seasonally-changing ornamental attributes, since observations and samples obtained at specific points in time can lead to flawed conclusions about their significance. Addressing the proximal causes of changes in plumage may provide more in-depth understanding of the links between inter-individual interactions, hormonal physiology and ornamental attributes.

## Supporting Information

Figure S1 **Boxplots and tests for initial morphology and color differences between treatment groups of male blue-black grassquits.**
(EPS)

## References

[1] Bortolotti GR (2006) Natural selection and coloration: protection, concealment, advertisement, or deception? In: Hill GE, McGraw KJ, editors. Bird Coloration Vol 2: Function and Evolution: Harvard University Press. 3–35.

[2] Andersson M (1994) Sexual Selection; Krebs J, Clutton-Brock T, editors. New Jersey, NJ: Princeton University Press. 599 p.

[3] Martinez-Padilla J, Mougeot F, Webster LMI, Pérez-Rodríguez L, Piertney SB (2010) Testing the interactive effects of testosterone and parasites on carotenoid-based ornamentation in a wild bird. Journal of Evolutionary Biology 23: 902–913. 20536879 10.1111/j.1420-9101.2010.01956.x

[4] Badyaev AV, Qvarnström A (2002) Putting sexual traits into the context of an organism: a life-history perspective in studies of sexual selection. The Auk 119: 301–310.

[5] Lozano G (1994) Carotenoids, parasites, and sexual selection. Oikos 70: 309–3011.

[6] Jouventin P, McGraw KJ, Morel M, Célerier A (2007) Dietary carotenoid supplementation affects orange beak but not foot coloration in Gentoo penguins *Pygoscelis papua* . Waterbirds 30: 573–578.

[7] Karubian J, Lindsay WR, Schwabl H, Webster MS (2011) Bill coloration, a flexible signal in a tropical passerine bird, is regulated by social environment and androgens. Animal Behaviour 81: 795–800.

[8] Fitze PS, Cote J, San-Jose LM, Meylan S, Isaksson C, et al (2009) Carotenoid-based colours reflect the stress response in the common lizard. PLoS ONE 4: e5111. 19352507 10.1371/journal.pone.0005111 PMC2663031

[9] Dijkstra P, Hekman R, Schulz Rd, Groothuis T (2007) Social stimulation, nuptial colouration, androgens and immunocompetence in a sexual dimorphic cichlid fish. Behavioral Ecology and Sociobiology 61: 599–609.

[10] von Schantz T, Bensch S, Grahn M, Hasselquist D, Wittzell H (1999) Good genes, oxidative stress and condition-dependent sexual signals. Proceedings of the Royal Society B: Biological Sciences 266: 1–12. 10081154 10.1098/rspb.1999.0597 PMC1689644

[11] Brawner WR, Hill GE, Sundermann CA (2000) Effects of coccidial and mycoplasmal infections on carotenoid-based plumage pigmentation in male house finches. The Auk 117: 952–963.

[12] Loiseau C, Sorci G, Dano SP, Chastel O (2008) Effects of experimental increase of corticosterone levels on begging behavior, immunity and parental provisioning rate in house sparrows. General and Comparative Endocrinology 155: 101–108. 17448473 10.1016/j.ygcen.2007.03.004

[13] Meylan S, Clobert J, Sinervo B (2007) Adaptive significance of maternal induction of density-dependent phenotypes. Oikos 116: 650–661.

[14] Doucet SM, Meadows MG (2009) Iridescence: a functional perspective. Journal of the Royal Society Interface 6 Suppl 2S115–132. 10.1098/rsif.2008.0395.focus PMC2706478 19336344

[15] Kinoshita S, Yoshioka S, Miyazaki J (2008) Physics of structural colors. Reports on Progress in Physics 71: 076401.

[16] Stoddard MC, Prum RO (2011) How colorful are birds? Evolution of the avian plumage color gamut. Behavioral Ecology 22: 1042–1052.

[17] Ghiradella HT, Butler MW (2009) Many variations on a few themes: a broader look at development of iridescent scales (and feathers). Journal of the Royal Society Interface 6: S243–S251. 10.1098/rsif.2008.0372.focus PMC2706475 19141432

[18] McGraw KJ, Mackillop E, Dale J, Hauber ME (2002) Different colors reveal different information: how nutritional stress affects the expression of melanin- and structurally based ornamental plumage. Journal Of Experimental Biology 205: 3747–3755. 12409501 10.1242/jeb.205.23.3747

[19] Prum R (2006) Anatomy, physics and evolution of structural colors. In: Hill G, McGraw K, editors. Bird Coloration Vol 1: Mechanisms and Measurements. Cambridge, MS: Harvard University Press. 295–353.

[20] Land MF (1972) The physics and biology of animal reflectors. Progress in biophysics and molecular biology 24: 75–106. 4581858 10.1016/0079-6107(72)90004-1

[21] Osorio D, Ham A (2002) Spectral reflectance and directional properties of structural coloration in bird plumage. Journal Of Experimental Biology 205: 2017–2027. 12089207 10.1242/jeb.205.14.2017

[22] Greenewalt C, Brandt W, Friel DD (1960) Iridescent colors of hummingbird feathers. Journal Of The Optical Society Of America 50: 1005–1013.

[23] Dufresne ER, Noh H, Saranathan V, Mochrie SGJ, Cao H, et al (2009) Self-assembly of amorphous biophotonic nanostructures by phase separation. Soft Matter 5: 1792–1795.

[24] Prum RO, Dufresne ER, Quinn T, Waters K (2009) Development of colour-producing beta-keratin nanostructures in avian feather barbs. Journal of the Royal Society Interface 6: S253–265. 10.1098/rsif.2008.0466.focus PMC2706469 19336345

[25] Maia R, Macedo RHF, Shawkey MD (2011) Nanostructural self-assembly of iridescent feather barbules through depletion attraction of melanosomes during keratinization. Journal of the Royal Society Interface. 10.1098/rsif.2011.0456 PMC3284138 21865251

[26] Fitzpatrick S (1998) Colour schemes for birds: structural coloration and signals of quality in feathers. Annales Zoologici Fennici 35: 67–77.

[27] Keyser AJ, Hill GE (1999) Condition-dependent variation in the blue-ultraviolet coloration of a structurally based plumage ornament. Proceedings of the Royal Society B: Biological Sciences 266: 771–777.

[28] Doucet SM (2002) Structural plumage coloration, male body size, and condition in the blue-black grassquit. The Condor 104: 30–38.

[29] Hill GE, Doucet SM, Buchholz R (2005) The effect of coccidial infection on iridescent plumage coloration in wild turkeys. Animal Behaviour 69: 387–394.

[30] Siefferman LM, Hill GE (2007) The effect of rearing environment on blue structural coloration of eastern bluebirds (*Sialia sialis*). Behavioral Ecology and Sociobiology 61: 1839–1846. 19655039 10.1007/s00265-007-0416-0 PMC2719904

[31] Peters A, Delhey K, Johnsen A, Kempenaers B (2007) The condition-dependent development of carotenoid-based and structural plumage in nestling blue tits: males and females differ. American Naturalist 169: S122–S136. 10.1086/510139 29517928

[32] Peters A, Astheimer LB, Boland C, Cockburn A (2000) Testosterone is involved in acquisition and maintenance of sexually selected male plumage in superb fairy-wrens, *Malurus cyaneus* . Behavioral Ecology and Sociobiology 47: 438–445.

[33] Collis K, Borgia G (1993) The costs of male display and delayed plumage maturation in the satin bowerbird (*Ptilonorhynchus violaceus*). Ethology 94: 59–71.

[34] Doucet SM, Montgomerie R (2003) Multiple sexual ornaments in satin bowerbirds: ultraviolet plumage and bowers signal different aspects of male quality. Behavioral Ecology 14: 503–509.

[35] Costa F, Macedo RH (2005) Coccidian oocyst parasitism in the blue-black grassquit: influence on secondary sex ornaments and body condition. Animal Behaviour 70: 1401–1409.

[36] Shawkey MD, Pillai SR, Hill GE, Siefferman LM, Roberts SR (2007) Bacteria as an agent for change in structural plumage color: correlational and experimental evidence. American Naturalist 169: S112–S121. 10.1086/510100 19426087

[37] Peters A, Kurvers RHJM, Roberts ML, Delhey K (2011) No evidence for general condition-dependence of structural plumage colour in blue tits: an experiment. Journal of Evolutionary Biology 24: 976–987. 21306464 10.1111/j.1420-9101.2011.02229.x

[38] Rubenstein DR, Hauber ME (2008) Dynamic feedback between phenotype and physiology in sexually selected traits. Trends in Ecology and Evolution 23: 655–658. 18951654 10.1016/j.tree.2008.07.010

[39] Sick H (1997) Ornitologia Brasileira. Rio de Janeiro: Editora Nova Fronteira. 862 p.

[40] Alderton C (1963) The breeding behavior of the blue-black grassquit. The Condor: 154–162.

[41] Maia R, Macedo RH (2011) Achieving luster: prenuptial molt pattern predicts iridescent structural coloration in blue-black grassquits. Journal of Ornithology 152: 243–252.

[42] Webber T (1985) Songs, displays, and other behavior at a courtship gathering of blue-black grassquits. The Condor: 543–546.

[43] Almeida J, Macedo RH (2001) Lek-like mating system of the monogamous blue-black grassquit. The Auk 118: 404–411.

[44] Carvalho CBV, Macedo RH, Graves JA (2006) Breeding strategies of a socially monogamous neotropical passerine: extra-pair fertilizations, behavior, and morphology. The Condor 108: 579–590.

[45] Carvalho CBV, Macedo RH, Graves JA (2007) Reproduction of blue-black grassquits in central Brazil. Brazilian Journal of Biology 67: 275–281. 10.1590/s1519-69842007000200012 17876437

[46] Dias RI, Kuhlmann M, Lourenco LR, Macedo RH (2009) Territorial clustering in the blue-black grassquit: reproductive strategy in response to habitat and food requirements? The Condor 111: 706–714.

[47] Dias R, Santos E, Macedo RH (2009) Mating system and sexual conflict in the blue-black grassquit (*Volatinia jacarina*, Aves:Emberizidae): extra-pair mating behavior sets the scene. Oecologia Brasiliensis 13: 183–191.

[48] Maia R, Caetano JVO, Báo SN, Macedo RH (2009) Iridescent structural colour production in male blue-black grassquit feather barbules: the role of keratin and melanin. Journal of the Royal Society Interface 6: S203–S211. 10.1098/rsif.2008.0460.focus PMC2706474 19141431

[49] Shawkey MD, Hill GE (2006) Significance of a basal melanin layer to production of non-iridescent structural plumage color: evidence from an amelanotic Steller's jay (*Cyanocitta stelleri*). Journal of Experimental Biology 209: 1245–1250. 16547296 10.1242/jeb.02115

[50] Lacava RV, Brasileiro L, Maia R, Oliveira RF, Macedo RH (2011) Social environment affects testosterone level in captive male blue-black grassquits. Hormones and Behavior 59: 51–55. 20950617 10.1016/j.yhbeh.2010.10.003

[51] Aguilar TM, Maia R, Santos ESA, Macedo RH (2008) Parasite levels in blue-black grassquits correlate with male displays but not female mate preference. Behavioral Ecology 19: 292–301.

[52] Rasband W (1997–2011) ImageJ. Bethesda, MD: National Institutes of Health.

[53] Finger E, Burkhardt D (1994) Biological aspects of bird colouration and avian colour vision including ultraviolet range. Vision research 34: 1509–1514. 8023462 10.1016/0042-6989(94)90152-x

[54] Hart NS, Partridge JC, Cuthill IC, Bennett ATD (2000) Visual pigments, oil droplets, ocular media and cone photoreceptor distribution in two species of passerine bird: the blue tit (*Parus caeruleus* L.) and the blackbird (*Turdus merula* L.). Journal of Comparative Physiology A: Sensory, Neural and Behavioral Physiology 186: 375–387. 10.1007/s003590050437 10798725

[55] Doucet SM, Mennill DJ, Montgomerie R, Boag PT, Ratcliffe LM (2005) Achromatic plumage reflectance predicts reproductive success in male black-capped chickadees. Behavioral Ecology 16: 218–222.

[56] Hunt S, Bennett ATD, Cuthill IC, Griffiths R (1998) Blue tits are ultraviolet tits. Proceedings of the Royal Society B: Biological Sciences 265: 451–455.

[57] Cuthill IC, Bennett ATD, Partridge JC, Maier E (1999) Plumage reflectance and the objective assessment of avian sexual dichromatism. American Naturalist 153: 183–200. 10.1086/303160 29578758

[58] Montgomerie R (2006) Analyzing colors. In: Hill G, McGraw K, editors. Bird Coloration. Cambridge, MS: Harvard University Press. 90–147.

[59] Wood SN (2006) Generalized additive models: an introduction with R: Chapman & Hall/CRC.

[60] Hastie T, Tibshirani R (1993) Varying-coefficients model. Journal of the Royal Statistical Society Series B: Statistical Methodology 55: 757–796.

[61] R Development Core Team (2012) R: A Language and Environment for Statistical Computing. Vienna, Austria: R Foundation for Statistical Computing.

[62] Kimball RT (2006) Hormonal control of coloration. In: Hill G, McGraw K, editors. Bird Coloration. Cambridge, MS: Harvard University Press. 431–468.

[63] Santos ESA, Maia R, Macedo RH (2009) Condition-dependent resource value affects male-male competition in the blue-black grassquit. Behavioral Ecology 20: 553–559.

[64] Doucet SM, Shawkey MD, Hill GE, Montgomerie R (2006) Iridescent plumage in satin bowerbirds: structure, mechanisms and nanostructural predictors of individual variation in colour. Journal Of Experimental Biology 209: 380–390. 16391360 10.1242/jeb.01988

[65] Folstad I, Karter A (1992) Parasites, bright males, and the immunocompetence handicap. American Naturalist: 603–622.

[66] Muñoz A, Aparicio JM, Bonal R (2008) Male barn swallows use different resource allocation rules to produce ornamental tail feathers. Behavioral Ecology 19: 404–409.

[67] Gosler AG (1994) Mass-change during moult in the great tit *Parus major* . Bird Study 41: 146–154.

[68] Senar JC, Domènech J, Uribe F (2002) great tits (*Parus major*) reduce body mass in response to wing area reduction: a field experiment. Behavioral Ecology 13: 725–727.

[69] Dietz MW, Daan S, Masman D (1992) Energy requirements for molt in the kestrel *Falco tinnunculus* . Physiological Zoology 65: 1217–1235.

[70] Klaassen M (1995) Moult and basal metabolic costs in males of two subspecies of stonechats: the European *Saxicola torquata rubicula* and the East African *S. t. axillaris* . Oecologia 104: 424–432. 28307657 10.1007/BF00341339

[71] Endler J, Théry M (1996) Interacting effects of lek placement, display behavior, ambient light, and color patterns in three neotropical forest-dwelling birds. American Naturalist 148: 421–452.

[72] Osorio D, Miklósi A, Gonda Z (1999) Visual ecology and perception of coloration patterns by domestic chicks. Evolutionary Ecology 13: 673–689.

[73] Galván I, Sanz JJ (2008) The cheek plumage patch is an amplifier of dominance in great tits. Biology Letters 4: 12–15. 18029295 10.1098/rsbl.2007.0504 PMC2412933

[74] Galván I (2008) The importance of white on black: unmelanized plumage proportion predicts display complexity in birds. Behavioral Ecology and Sociobiology 63: 303–311.

